# Adult Onset Morgagni Hernia: Medical vs. Surgical Management

**DOI:** 10.7759/cureus.4626

**Published:** 2019-05-09

**Authors:** Ahmed Elfiky, Danial Daneshvar, Michael Krzyzak, Indraneil Mukherjee

**Affiliations:** 1 Internal Medicine, Northwell Health-Staten Island University Hospital, New York, USA; 2 Surgery, Northwell Health-Staten Island University Hospital, New York, USA

**Keywords:** morgagni hernia

## Abstract

Morgagni hernia is a type of diaphragmatic hernia where bowel content herniates through an irregular opening into the thoracic cavity. Herein, we present the case of an 84-year-old female patient with multiple hospital admissions for abdominal symptoms. Radiological studies confirmed Morgagni hernia. She underwent a laparoscopic intervention with mesh placement. She was discharged in stable condition and was doing well on follow-up.

## Introduction

Morgagni hernia is a type of diaphragmatic hernia where the bowel content herniates through an irregular opening first described in 1769 by Italian anatomist and pathologist Giovanni Battista Morgagni (February 25, 1682 - December 6, 1771) ​​​​who was generally regarded as the father of modern anatomical pathology. Morgagni hernias constitute only 2% to 4% of congenital diaphragmatic hernias and symptomatic adult cases are extremely rare [1]. Morgagni hernias are generally asymptomatic and frequently found incidentally during routine diagnostic tests for other reasons. Rarely, they may present with intestinal obstruction or respiratory symptoms [2].

The defect involves a failure of the fusion of the septum transversum, the diaphragm, and the costal arches. Even though the Morgagni opening is often congenital, conditions such as pregnancy, trauma, chronic cough, obesity, and constipation, all of which may increase the intra-abdominal pressure, predispose to the development of the condition [3].

## Case presentation

**Figure 1:**
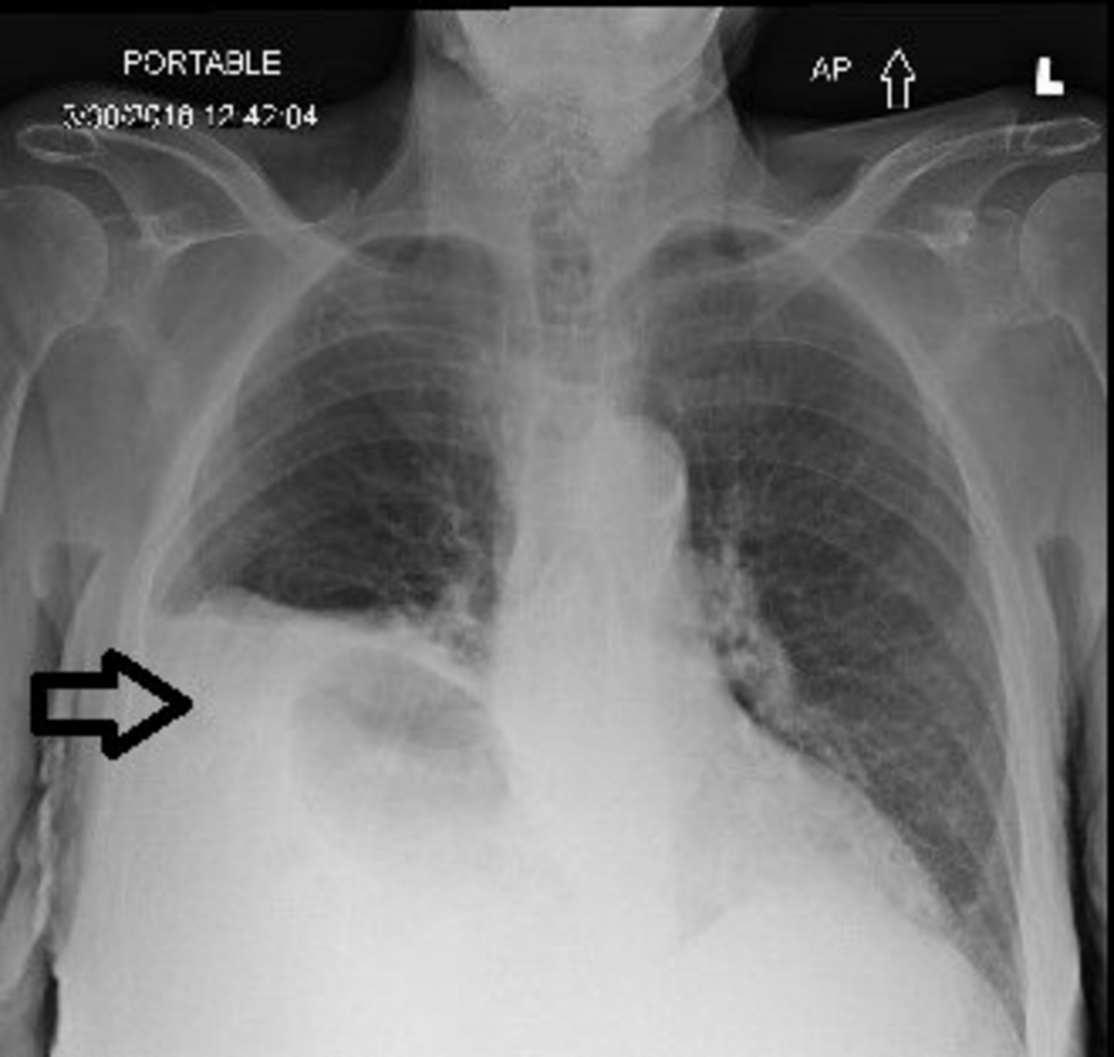
Radiograph of chest anterior-posterior view; arrow indicates the elevation of the right hemidiaphragm with diaphragmatic hernia

An 84-year-old female with no prior history of surgery or trauma presented to the hospital with nausea, vomiting, and abdominal pain for three days. At the time of the presentation, the patient was hemodynamically stable. X-ray of the chest showed the elevation of the right hemidiaphragm with right basilar atelectasis suggestive of diaphragmatic hernia (Figure 1).

**Figure 2:**
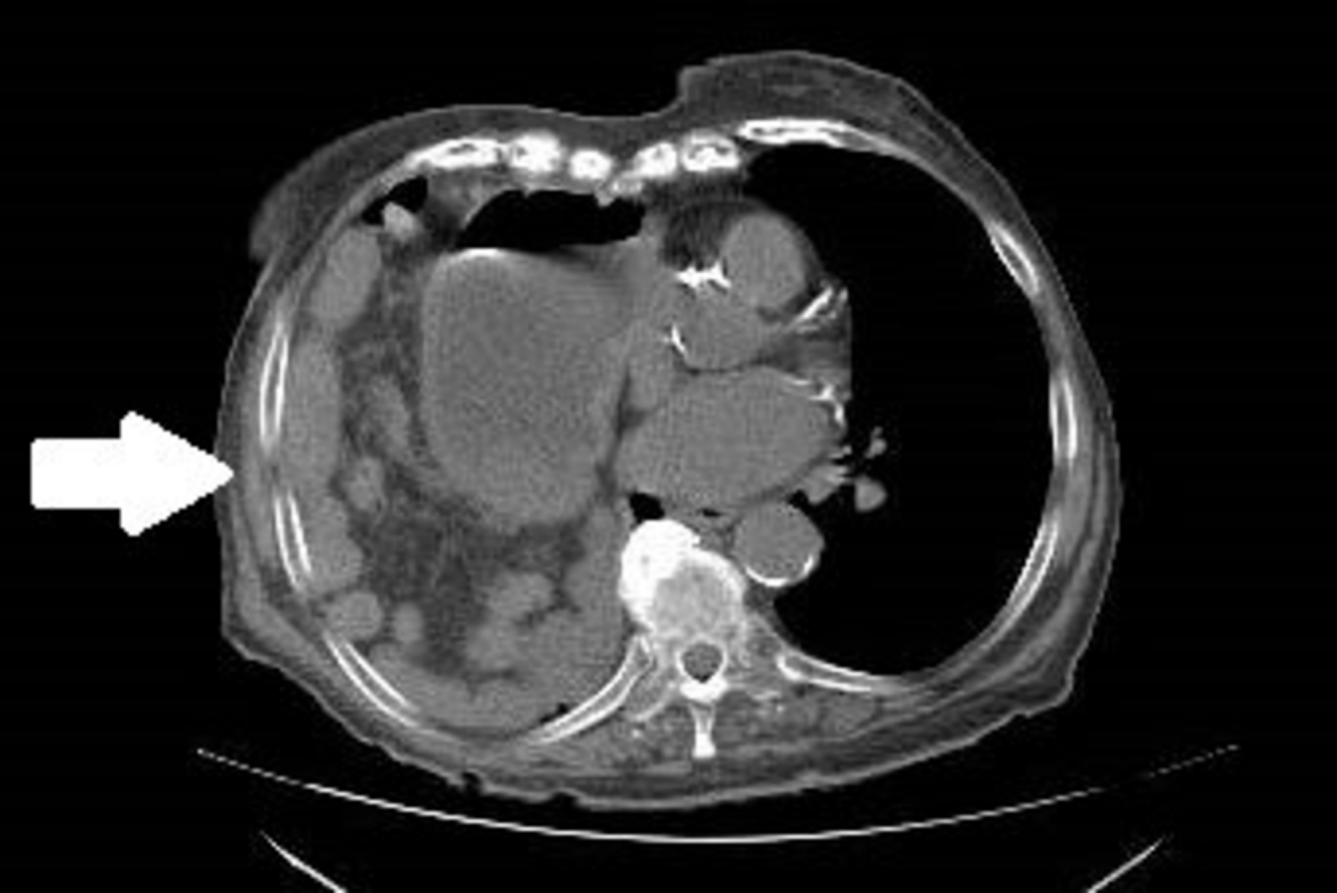
Axial view computed tomography on admission; arrow indicating stomach and intestinal contents in the thoracic cavity

**Figure 3:**
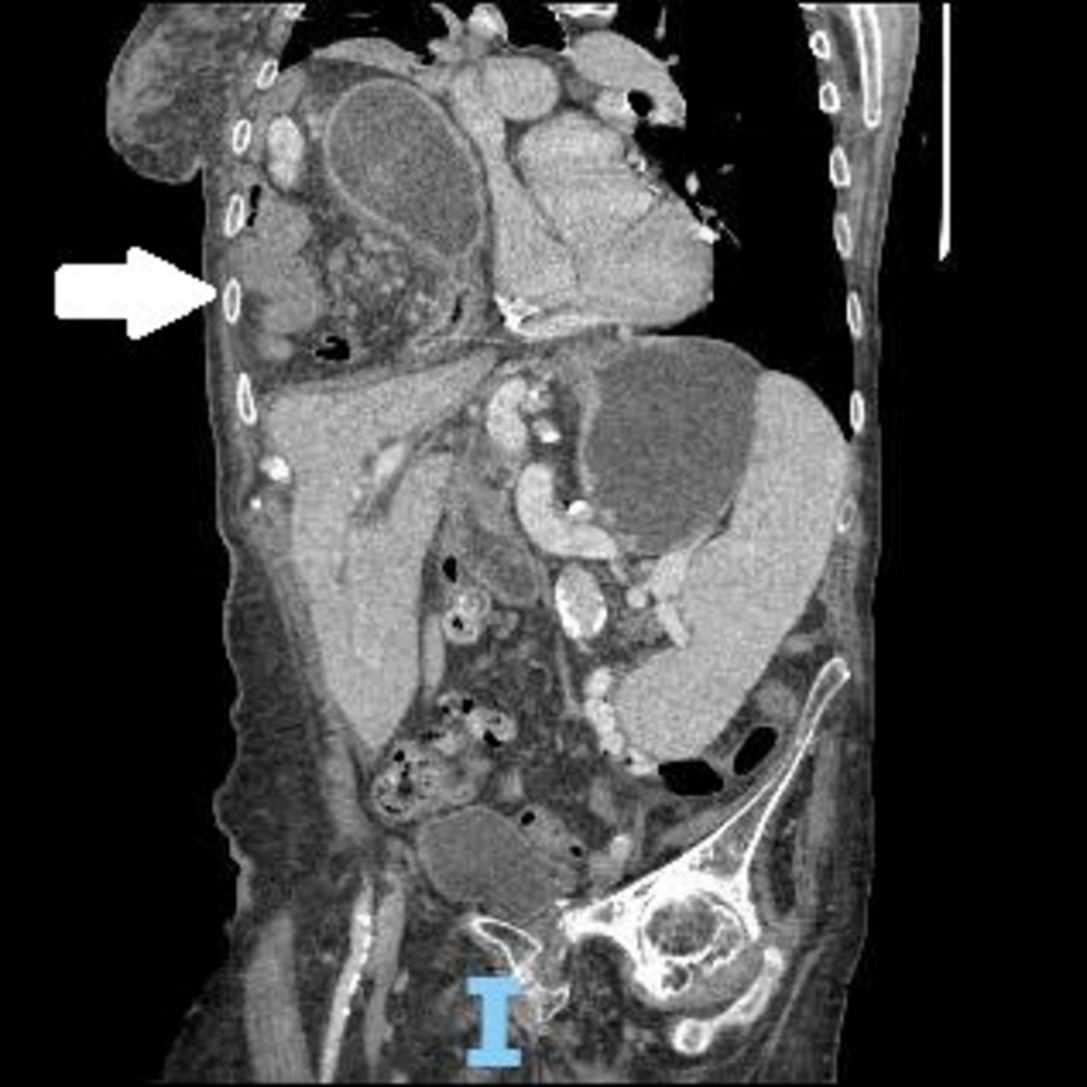
Coronal view computed tomography on admission; arrow indicating stomach and intestinal contents in the thoracic cavity

The CT scan confirmed the diagnosis of diaphragmatic hernia containing loops of small bowel and distal stomach classified as a large right Morgagni hernia (Figures 2-3).

The patient was seen by a gastroenterologist and surgeon. Based on her age and other comorbidities, including her history of recent pulmonary embolism, the patient was considered high risk for surgical intervention. The decision was made to proceed with medical management, including intravenous fluid and nasogastric tube drainage, which resulted in the resolution of the symptoms over a period of 10 days and the patient was discharged home on pantoprazole and sucralfate with a regular diet.

The patient presented one month later to the emergency department with similar symptoms. Repeat radiologic studies showed a stable, large, right-sided Morgagni hernia. Shortly after the admission, and due to recurrent symptoms leading to poor quality of life, the decision was made to proceed with surgical intervention and the Morgagni hernia was repaired and mesh placed using laparoscopic approach to prevent recurrence.

**Figure 4:**
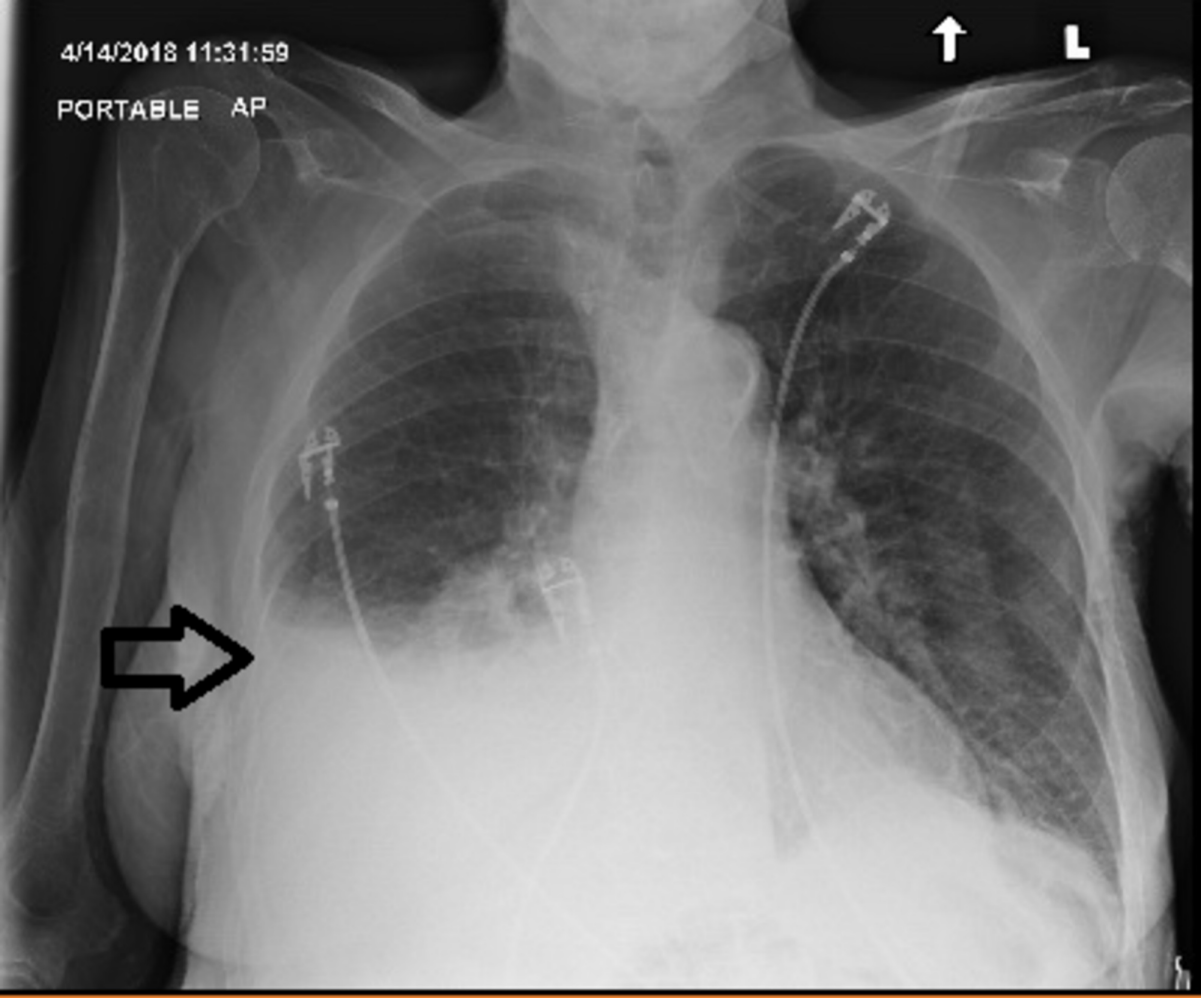
Radiograph of chest anterior-posterior view; arrow indicates the postoperative pleural fluid collection expected after surgical correction of the diaphragmatic hernia

The patient tolerated the procedure well. A postoperative chest X-ray showed only a small, right-sided pleural effusion without evidence of the previous hernia (Figure 4).

She was discharged three days after the surgery and was completely asymptomatic three months after the surgery, tolerating a regular diet.

## Discussion

Morgagni hernia is a congenital defect that usually presents in adulthood. The congenital weakness in the diaphragm is usually small; as the patient becomes older, the defect enlarges secondary to increased intra-abdominal pressure. It is generally asymptomatic [4]. It can also present with intestinal obstruction or respiratory distress. Since these symptoms are common, high clinical awareness is warranted to diagnose this rare condition.

Surgery is the treatment of choice for Morgagni hernias. The two main surgical approaches have been described for the treatment of these hernias: transabdominal (open or laparoscopic) and transthoracic (open or thoracoscopic) [5].

The first laparoscopic repair of a Morgagni hernia was reported by Küster in 1992 and has since gained significant popularity [6], but the laparotomy approach has been more reported.

The abdominal approach allows for the easier reduction of the hernia contents, evaluation of the contralateral diaphragm for additional defects, and the concomitant evaluation and repair of intraabdominal pathology.

Reviewing the literature for the treatment of Morgagni hernia in adults reveals a major question regarding the closure of the defect, whether the defect should be closed primarily, and/or the use of mesh for bridging or reinforcement. Out of 46 laparoscopic cases reported in the literature, primary closure of the defect is described in 29% of the cases, and mesh placement in 7% of the cases. [7]

In our case, conservative management resulted only in the temporary resolution of the symptoms and the patient underwent laparoscopic repair subsequently as the gold standard treatment of Morgagni hernia. Mesh is also used during the procedure to provide the tension-free surface required for the optimum closure especially with a large-sized defect.

Few cases of robotic surgery of Morgagni hernia repair have been reported [7]. There is not enough evidence for the robotic surgery to be the gold standard procedure for these types of hernia at this time. Every case should be evaluated on an individual basis on which approach is best for the patient.

## Conclusions

The definite treatment of Morgagni hernia is warranted even in asymptomatic cases to prevent life-threatening complications such as intestinal obstruction and peritonitis. The laparoscopic approach is considered the leading modality that results in symptom resolution and improvement in the quality of life with minimal side effects and short postoperative stay, even in the elderly population.
